# Establishing Cell Lines from Fresh or Cryopreserved Tissue from the Great Crested Newt (*Triturus cristatus*): A Preliminary Protocol

**DOI:** 10.3390/ani11020367

**Published:** 2021-02-01

**Authors:** Julie Strand, Henrik Callesen, Cino Pertoldi, Stig Purup

**Affiliations:** 1 Department of Chemistry and Bioscience, Aalborg University, Fredrik Bajers Vej 7K, DK-9220 Aalborg Øst, Denmark; cp@bio.aau.dk; 2 Department of Animal Science, Aarhus University, Blichers Allé 20, DK-8830 Tjele, Denmark; henrik.callesen@anis.au.dk (H.C.); stig.purup@anis.au.dk (S.P.); 3 Aalborg Zoo, Mølleparkvej 63, DK-9000 Aalborg, Denmark

**Keywords:** cell culture, morphology, culture conditions, xCELLigence^®^, real-time cell analysis, amphibian, cryopreservation, biobanking

## Abstract

**Simple Summary:**

This research describes a successful protocol for establishing viable cell lines from one of the amphibian species that are severely threatened with extinction. For the study, fresh or cryopreserved tissue samples were used, and various parameters affecting the optimal growth conditions were tested, such as use of different media, impact of media change and the influence of contamination. We concluded that: (i) Using fresh tissue was more successful in terms of establishing cell lines; (ii) No differences were found between the media used for fresh tissue, while one medium had a positive effect on growth when using cryopreserved tissue; (iii) Contamination caused the tissue cultured to be destroyed; and (iv) Real-time cell analysis visualized a significant impact on cell growth pattern when media change was combined with PBS washing. This research demonstrated the potential of and the challenges to establishing amphibian cell lines, which provide an opportunity for implementing modern reproductive technologies as a future part of conservation strategies for endangered and threatened amphibian species.

**Abstract:**

This study describes a successful protocol for establishing cell lines from the threatened *Triturus cristatus* in terms of collection, preparing, establishing, cryopreserving, thawing and quality checking. Different parameters such as media, media change, fresh vs. cryopreserved tissue and seeding density were tested to optimize culture conditions for this species. With fresh tissue, no considerable differences in the use of two different media were found, but with cryopreserved tissue, a combination of ITS (insulin/transferrin/selenite) and 2-mercaptoethanol had a positive effect on growth. Real-time measurements on the cell lines were used, for the first time in amphibian cells, to investigate the effect of different treatments such as media change with or without washing. Media change had a positive impact on the cells, whereas the effect was negative when combined with washing. It is concluded that establishment of cell lines is possible from the great crested newt, especially when using fresh tissue, but much more challenging if the tissue has been cryopreserved. Real-time measurement during cell culture is a useful tool to visualize the sensitivity of amphibian cells during different culture treatments.

## 1. Introduction

The great crested newt (*Triturus cristatus*) has suffered severe declines mainly due to habitat destruction, habitat degradation, pollution, fragmentation, introduction of fish and lack of pond management [1]. These changes are observed globally among many amphibian species, and the number of species at risk of extinction continues to grow [2,3]. Therefore, various conservation strategies and efforts have been taken in situ, but due to consistent anthropogenic pressures, ex situ strategies such as biobanking are becoming essential for future species conservation [3].

Biobanking combined with cryopreservation and modern reproductive technologies are becoming a vital addition to conservation strategies of amphibians and other animal groups. Cryopreservation provides an optimal technique for long-term storage of genetic materials as the biological, chemical and physical processes are suspended due to the low temperatures [4]. Cell lines grown from tissue samples taken postmortem or via non-lethal techniques are a supplement to biobanking, which has already proven useful in relation to conservation in other vertebrate species such as the African elephant (*Loxodonta Africana)*, the clouded leopard (*Neofelis nebulosi)* and the western lowland gorilla (*Gorilla gorilla gorilla)* [3,5,6]. Stored biological material provides opportunities for species-specific stem cell technologies (induced pluripotent stem cells), collection of gametes, production of artificial gametes, in vitro embryo production and embryo transfer [3,7], but use of amphibian cell lines in conservation strategies is still very limited due to lack of basic knowledge [2]. Cryopreserved genetic material and especially cell lines provide several opportunities, and therefore it is important to ensure the safeguarding of these valuable resources as long-term storage makes it possible to utilize the samples for techniques that have not yet been invented.

A considerable number of studies have been reported on cultivation of amphibian cells from various species of embryos, tadpoles and adults. However, research on amphibian cell lines is limited to very few families and species, and most of these studies are more than 20 years old [8,9,10,11,12].

No biobanked cell lines have been reported from the great crested newt (*Triturus cristatus*), and the aim of this study was therefore to develop and validate a protocol for establishing cell lines within this species. We succeeded in establishing cell lines from two out of four animals and therefore report our preliminary protocol to collect, prepare, establish, cryopreserve, thaw and quality check cell lines. Various parameters were evaluated for their effect on growth conditions, such as cryopreserved versus fresh tissue samples, different treatment media and impact of media change. Likewise, we demonstrated the first reported use of real-time cell analysis on amphibian cell lines and discuss the potential value of this analysis in future work.

## 2. Methods

### 2.1. Biopsy and Culture Media

Biopsy medium was prepared according to [13]: completed Alpha MEM media supplemented with 1% antibiotic-antimycotic 100X (P/S/F) (10,000 units/mL of penicillin 10,000 µg/mL of streptomycin and 25 µg/mL of Fungizone (Amphotericin B)) (Gibco^®^, Life Technologies, Rockville, MD, USA).

Culture media (see Table 1 for details): one medium was tested with three different supplements. The original study design only included media A and B, but due to parallel running studies with cryopreserved tissue explants, we added media C and D to test the effect of the reducing agent 2-mercaptoethanol on tissue Life Technologies, Rockville, MD, USA cultures of cryopreserved explants.

### 2.2. Pre-Treatment and Primary Cell Culture

Tissue from hind legs was used for this experiment, as this is one of the recommended tissue types for establishing amphibian cell lines [4,13]. Immediately after euthanasia the experimental tissues were obtained from the hind legs after wiping with 70% *v/v* ethanol and washing in Dulbecco’s phosphate-buffered saline (+ 1000 mg/L CaCl_2_ and 1000 mg/L MgCl_2_) (PBS) (Gibco^®^ Life Technologies, Rockville, MD, USA) (this PBS was used throughout the study). Tissues were stored in biopsy media at 4 °C before further processing. Half of the tissues were cryopreserved with 10% DMSO as cryoprotectant, and cryovials (Nunc^®^, Thermo Scientific, Roskilde, Denmark) were placed in a CoolCell^®^ (Sigma) and stored at −80 °C for 24 h before being transferred to liquid nitrogen (LN_2_) [13]. Fresh tissues (approximately 0.5 mm^3^ in size) were minced with scalpel and forceps [13]. The tissue was hereafter immersed in 70% *v/v* ethanol for 30 s, washed three times with PBS, immersed in antibiotic–antimycotic for 20 s, washed once in PBS, immersed in gentamicin (Sigma-Aldrich, Inc, St.Louis, MO, USA) for 20 s and then washed in PBS. The minced tissues were placed on the backside of a 12.5 cm^2^ cell culture flask and left until they were attached (3–6 min) before adding 2 mL of one of media A–D. All individuals were set up with 3 replicates by the tissue explant method [13]. After 16 weeks the cryopreserved tissue was thawed in a 30 °C water bath for 1–2 min and processed as described above. The cell cultures were maintained at 28 °C with 5% CO_2_ with the medium being changed every three to four days. Primary cells appeared on average on days 22 and 39 from fresh and cryopreserved tissue, respectively. At 80% confluency, cells were passaged, i.e., harvested by adding 0.5 mL of TrypLE (Gibco^®^ Life Technologies, Rockville, MD, USA) and thereafter split into new cell culture flasks at a ratio of 1:2–1:3 to be incubated under the same conditions as above.

### 2.3. Cryopreservation and Recovery

After six passages the cells were harvested by adding 1.5 mL TrypLE (Gibco^®^ Life Technologies, Rockville, MD, USA). After all cells were detached, 8 mL Hanks Balanced Salt Solution (1X) (HBSS) (Gibco^®^ Life Technologies, Rockville, MD, USA) was used to rinse all cells before transfer to a 15 mL centrifuge tube. Cells were centrifuged for 10 min at 300 × G, the supernatant was removed, and the cell pellet resuspended in freezing medium (Alpha MEM media supplemented with 10% DMSO). Cell suspensions were kept in cryovials (Nunc^®^) and placed in a CoolCell^®^ (Sigma-Aldrich, Inc., St. Louis, MO, USA) at −80 °C for 24 h before transfer to LN_2_. For recovery of cell suspensions, frozen vials were thawed in a 30 °C water bath for 1–2 min, and then transferred into 25 cm^2^ cell culture flasks containing 4 mL of completed media and cultured at 28 °C. See Table 1 for specific media descriptions.

### 2.4. Evaluation of Proliferation and Growth Conditions

Real-time measurements of cell proliferation and adhesion were performed on thawed cell lines using xCELLigence^®^ real-time cell analysis (RTCA SP Bundle, ACEA Biosciences), according to the instruction manual. This system consists of an electronic sensor analyzer with gold electrodes on the bottom of the wells collecting impedance readings that are converted into a cell index (CI). Different cell types reveal different CI, which provide an indication of adhesion and proliferation rates. Non-adherent cells will not affect the CI values. The CI is a relative and dimensionless value that represents the change in impedance divided by the background value [14,15]. Throughout the text, CI will be described as CI/h to identify CI at a specific time. In brief, thawed cell lines were seeded in 25 cm^2^ cell culture flasks (Nunc^®^ Easy flask, Roskilde, Denmark) containing 4 mL of completed media and incubated at 28 ℃. To prepare cells for seeding, they were detached after passage 3 using 1.5 mL TrypLE (Gibco^®^ Life Technologies, Rockville, MD, USA) for 1 min. at 28 °C. To block the enzyme activity, media A–D was used and hereafter cell suspensions were counted with the Countess^TM^ II FL (Applied Biosystems). Two different cell lines from two different individuals were seeded in four replicates with the following seeding densities; 5000 cells/well, 7500 cells/well and 10,000 cells/well. Three different medium treatments were tested: no media change (NoMC), media change (MC) and media change including 10 s washing with PBS (MCPBS). After seeding, the plate was incubated at 28 °C with 5% CO_2_. The medium was changed every 72 h. The impedance readings were continually measured every 30 min and displayed as the CI. Control wells were included and contained only DMEM culture medium and no cells, and they all displayed zero CI values. Data acquisition and analysis were performed with the RTCA software (Agilent, Santa Clara, CA, USA).

### 2.5. Animals

Four wild caught individuals of *Triturus cristatus* were used for the experiment. To collect this material, a dispensation from Act nr. 1466 (Protection of Species) was given by the Danish Ministry of Environment and Food of Denmark (J.nr. MST-850-00110). All experiments complied with the EU Directive 2010/63/EU for Animal Experiments.

### 2.6. Statistical Analysis

Differences in mean cell index values among cell number and treatments were assessed with one-way ANOVAs whereafter Tukey’s test was used to test the difference within treatments. T-tests were used to detect significant differences between means when using either fresh or cryopreserved tissue. The software program PAST was used for all statistical analysis: https://folk.uio.no/ohammer/past/.

## 3. Results

### Development of Cell Lines

Cell lines were established from two and one of the four individuals from either fresh or cryopreserved tissue, respectively.

**Figure 1:**
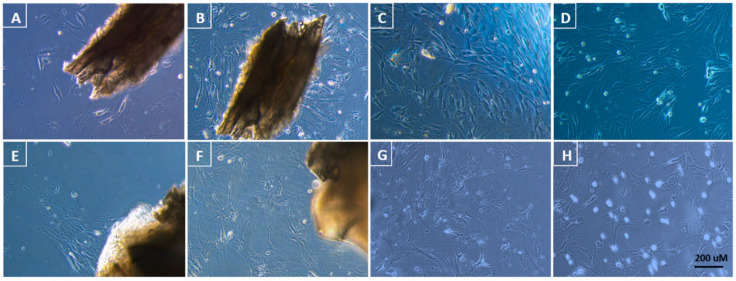
Tissue culture of *Triturus cristatus*: (**A**) primary culture of fresh tissue at day 30; (**B**) day 35; (**C**) passage 2 cells pre-cryopreservation; (**D**) passage 2 cells post-warming; (**E**) primary culture of cryopreserved tissue at day 45; (**F**) day 65; (**G**) passage 2 cells pre-cryopreservation; (**H**) passage 2 cells post-warming. 200 × magnification. Reference bar = 200 uM.

Cell appearance (Figure 1).

Cells were categorized based on morphological characteristics and are named as such throughout this study. Post-thaw viability was observed in all cell lines by daily observations of cell proliferation and adhesion. Figure 1 displays and describes cells from both fresh tissue as well as cryopreserved tissue pre-cryopreservation and post-thawing. Cryopreservation had no impact on proliferation patterns of any of the cell lines.

Fresh tissue: Primary cultures displayed a mix of epithelial star-like cells and spindle-shaped fibroblast-like cells, where fibroblast-like cells generally dominated the cultures (A–B). After two passages, most cells were gradually replaced by spindle-shaped fibroblast-like cells (C). After cryopreservation of the cell line, cells displayed similar spindle-shaped fibroblast-like cells as pre-cryopreservation (D).

Cryopreserved tissue: Primary cultures also showed a mix of epithelial- and fibroblast-like cells (E–F), but these cells displayed extensive dendritic activity forming a web-like constitution of cells. After passage 2, cultures were dominated by elongated web-like fibroblast cells with few star-like epithelial cells mixed in between (G). Elongated spindle-shaped fibroblast-like protruding dendritic processes were also found throughout passages. After cryopreservation of the cell line, similar elongated web-like fibroblasts were observed (H).

Growth of cells in different culture media is described in (Table 2).

Growth patterns were grouped as follows: (1) no cell growth within 14 weeks; (2) fungus was detected and treated but samples were not salvageable; (3) bacteria were detected and treated but samples were not salvageable; (4) cell growth appeared ranging from few to 50–100 cells, but growth then stopped; (5) cell lines were passaged at least six times before they were cryopreserved.

Fresh tissue: Cell growth was observed in 18 of 24 replicates. Among the 24 replicates, 12 were lost due to fungus and two due to bacteria. Three replicates from two different individuals reached the cell line stage (passaged at least six times) and were cryopreserved.

Cryopreserved tissue: Cell growth was observed in 13 of 48 replicates. Among the 48 replicates, 22 were lost due to fungus and four due to bacteria. One replicate reached the cell line stage and was cryopreserved.

Table S1 in Supplementary material displays parameters on the initial growth patterns observed in fresh and cryopreserved tissue, including replicates among the four different media. From fresh tissue, emerging cells were observed between days 3 and 67 and 3 and 78 (medium A and B, respectively), depending on the individual animal; however, in many replicates, cells never proliferated sufficiently to reach 80% confluency and thus passage 1. The average times from cell culture initiation to passage 1 and cryopreservation of cell lines were 56 and 119 days, respectively. From cryopreserved tissue, emerging cells were observed between days 28 and 36 (media A), day 72 (media B), days 35 and 40 (media C) and days 24 and 37 (media D). However, only one replicate reached the stage of passage 1 on day 58, and this cell line was cryopreserved on day 98.

Growth patterns during culturing.

**Figure 2:**
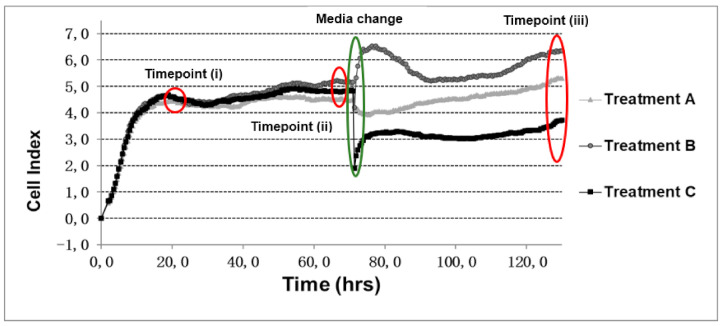
Model displaying the general growth pattern observed among treatments (**A**–**C**). Curves represent the mean cell index of four replicates.

The growth pattern observed throughout the 130 h of culture was divided into four different periods: (i) establishment, from 0–20 h; (ii) first growth period, from 20–70 h; (iii) media change at 72 h; (iv) second growth period, from 75–130 h. The general growth pattern observed in this study is displayed in Figure 2. To characterize each period, three time points were selected: (20 h (i), 70 h (ii) and 130 h (iii)). Furthermore, the change observed at media change was calculated as a percentage displaying a positive or negative impact.

Table 3 displays CI values of the three selected time points for individual 2. The growth patterns when using 10,000 cells reached a higher CI-level compared to both 7500 and 5000 cells at time point (i) (Supplementary material Figure S1). All treatments with 10,000 cells remained at this level until time point (ii), whereas growth patterns for both 7500 and 5000 cells decreased between the first two time points. The reaction to media change was seen as a drastic decrease in most treatments, but especially within treatment (C), where the decrease ranged between 44% and 87%. After the media change, all treatments reestablished their growth patterns until the last time point (iii), where most curves returned to the same CI level as before, except treatment (C), which displayed a noticeable lower CI level.

For individual 3, a similar growth pattern was observed throughout the treatments (Table 4). However, a difference in CI levels at time point (i) was observed as well as variations in CI level towards time point (ii) (Supplementary Material Figure S2). A similar drop in CI at media change was observed for most treatments, especially for treatment (C), where the drop ranged between 29% and 52%. After media change all treatments reestablished their growth patterns until the last time point (iii), when most curves displayed the same CI level as before media change, except for treatment (C), which displayed a noticeably lower CI level.

Differences among growth patterns were also tested for cells established by either fresh or cryopreserved tissue (Supplementary material Figure S3). As displayed in Table 5, a slower growth was seen at time point (i) when using fresh tissue, however a similar CI level was reached for cells from both tissue types at time point (ii). As seen previously, a drop in CI level was observed among cells from both tissue types after media change, especially for treatment (C), where a drastic drop was observed. Cells from fresh tissue displayed a drop ranging from 38% to 53% at first and second media change, respectively, whereas cells from cryopreserved tissue ranged from 47% to 48%, respectively. After media change all treatments reestablished their growth patterns until the last time point (iii), when most curves displayed the same CI level as before media change, except for treatment (C), which displayed a visibly lower CI level.

## 4. Discussion and Conclusion

### 4.1. Parameters Affecting the Success Rate of Amphibian Cell Lines

The tissue explant method was used for both fresh and cryopreserved tissue material, as described and recommended by Houck et al. [13]. This is the preferred method for establishing long-term cell cultures [16,17,18], whereas studies focusing on short-term primary cultures have used various enzyme digestions [8,19,20,21]. A distinctive difference between use of fresh and cryopreserved tissue was the high number of cryopreserved tissue explants where outgrowth from the explant was never observed. This could call for testing of other methods such as partial enzymatic digestion [22]. Contamination and lack of growth were the dominating factors affecting the success rate of the cell lines (Table 2), both fresh (14 out of 24 lost) and cryopreserved tissue cultures (26 out of 48 lost). When bacteria or fungus were detected, the manual cleansing technique applied could not salvage any of the samples. Previous studies have reported various attempts, with varying success rates, to reduce the amount of contamination including different antibiotics such as a combination of penicillin and streptomycin [17,23,24,25] or gentamicin solely [16] or in combination with other antibiotics [21,26]. We tried an additional washing step with 1% (v/v) Normocin for 20 s, as positive results were seen in parallel studies on other species in our lab [27]; however, the samples were still not salvageable. Future studies could determine the specific type of fungus and bacteria to find a more targeted treatment to avoid contamination. In addition, it has been recommended to keep wild-caught animals in captivity for several weeks to further reduce contamination [4].

### 4.2. Testing Optimal Growth Conditions

We tested medium with/without ITS on the fresh tissue explants, but due to parallel running studies in our lab with cryopreserved tissue explants in species of *Bufo bufo* and *Rana arvalis* [27], we also tested 2-mercaptoethanol even though similar experiments on fresh tissue would lack a control group.

In fresh tissue explant cultures from all four individuals, growth patterns did not show any considerable changes in terms of growth initiation using media A or B. However, the combination of ITS and 2-mecaptoethanol in media D indicated a positive effect on growth initiation of cryopreserved tissue explants. On average, growth initiated at days 22 and 39 for fresh and cryopreserved tissue, respectively. Other studies working with fresh tissue explant cultures of the white-lipped tree frog (*Litoria infrafrenata)* and the Chinese giant salamander (*Andrias davidianus)* reported initiated growth after 11 and 30 days, respectively [16,17]. Due to the slow growth rate of amphibian cells, 150 days was found to be the average culture time before having a sufficient number of cells to cryopreserve [4]; this corresponds with the average of 119 days in our study. No reports studying growth conditions of cryopreserved tissue explants from amphibians have been found, therefore no comparisons to our data have been possible. Overall, however, our results illustrate the importance of setting up cell cultures from fresh tissue if possible, as the success rate is significantly higher than when using cryopreserved material.

Real-time measurements of cell growth on amphibian cell lines are shown here for the first time, even though this technique is widely used; e.g., in humans, mice and rats [15,28,29,30,31,32]. It was therefore necessary to define the ideal seeding density for our species before conducting further experiments. Previous studies on vertebrate cells found 10,000 cells/well to be the optimum concentration for a dynamic monitoring of cell growth [28,29], so we chose seeding densities of 5000, 7500 and 10,000 cells/well. Based on our experiments we recommend working with amphibian cells at a density of 10,000 cells/well, as both individuals displayed better growth conditions and a moderate to strong CI when seeded with this density, compared to 5000 and 7500 cells/well [15]. However, more individuals need to be tested to determine variations between individuals from the same species. Moreover, variation is also expected between species, as methods and conditions successful for some amphibian species can fail to work on another species, even within the same genus [4].

Many cell culture protocols include a washing step with PBS or another physiological buffer (e.g., HBSS) during media change before cell dissociation or transporting of cells, so we tested if PBS had an effect on amphibian cells [32,33]. At media change, we observed a drop ranging from 44%–87% and 29%–52% in individuals 2 and 3, respectively. A simple media change (MC) did in general have a positive effect on growth patterns, indicating that the cells needed this change to get new supplies of nutrients, and the cells were not affected as profoundly as when adding a washing step with PBS. Media change with PBS washing had a significant and negative impact on all three seeding densities (Supplementary Figure S1 and S2), but at 130 h also resulted in a significantly lower CI level compared to those of cells with no media change and in most cases also media change (Table 3 and Table 4). Even though PBS is listed as a physiological buffer for cell dissociation, it has a significant impact on amphibian cells [33,34], so further studies are needed to evaluate another physiological buffer for washing, or to use, e.g., HBSS. Finally, when working with either fresh or cryopreserved tissue a significant difference was found after adhesion (20 h) and at the end of each treatment (130 h) (Table 5), indicating differences in growth patterns between the two tissues.

Taken together, the results demonstrate the usefulness of real-time cell measurements as a plausible addition for optimizing the limited knowledge on cell culture conditions for amphibians. However, the xCELLigence^®^ system is a very sensitive system, especially during media change, when treatments even without media change also displayed a drop in CI due to the associated temperature changes. However, our experiments have provided a real-time timeline of the adhesion and proliferation after different treatments of amphibian cell lines, which can be used to optimize the conditions for culture of other amphibian cell lines. Further research including various amphibian species and more individuals are, however, needed for comparisons in terms of testing and qualifying amphibian cell lines.

## 5. Conclusions

In the present study we report a preliminary biobanking protocol to collect, prepare, establish, cryopreserve, thaw and quality check cell lines from the great crested newt. We showed a higher success rate when using fresh compared with cryopreserved tissue. However, cryopreserved tissue can be used, but at a very low success rate. Contamination with either bacteria or fungus is a serious problem, as samples could not be salvaged after contamination had appeared. Further research is needed to reduce or even eliminate the degree of contamination and thereby the number of samples lost. We found no considerable differences when using media A and B on fresh tissue, but a positive effect was seen with cryopreserved tissue in media D. In future studies it would be interesting to see if the same positive effect will be found when working with fresh tissue. We demonstrated the first use of real-time cell measurements on amphibian cell lines, making it possible to examine adhesion and proliferation stages as well as the impact of different media treatments directly on the cells. Our general conclusions from using xCELLigence^®^ were: (i) A higher number of cells provided better establishment of the culture and gave a better indication of the growth pattern. (ii) Media change with PBS washing had a significant impact on the cell index at all seeding densities used. However, most cultures with a higher seeding density were able to reestablish, whereas growth of cultures with low seeding densities slowly decreased. (iii) Cells from cryopreserved tissue displayed a slower growth in the beginning as well as a reaction on media change, however afterwards they were thriving. (iv) Individual differences were seen among the cell lines.

Due to the current risk of extinction of amphibian species, action is needed. The use of cryopreserved cell lines in conservation provides opportunities for implementing modern reproductive technologies as a future part of conservation strategies for endangered and threatened amphibian species. However, to reach this point much more work is needed on establishing and culturing of amphibian cell lines.

## Figures and Tables

**Table 1:** Media composition including supplements used on fresh or cryopreserved tissue.

	Media	Supplements	Type of Tissue
**Medium A**	100% Cellgro Minimum Essential Medium (MEM) Alpha 1 X (Fisher Scientific) supplemented with 10% foetal bovine serum (Gibco Life Technologies, Rockville, MD, USA) and 1% penicillin-streptomycin–glutamine (29.2 mg/mL L-glutamine, 10,000 units/mL penicillin and 10,000 µg/mL streptomycin sulfate (Gibco^®^ Life Technologies, Rockville, MD, USA) [13]. Plus 0.1% Normocin (Invivogen 500 mg)		Fresh or cryopreserved
**Medium B**		20 µL/mL ITS (100 µL insulin 10 mg/mL + 100 µL transferrin 5.5 mg/mL + 10 µL selenite 20 µg/mL) (Sigma-Aldrich, Inc, St.Louis, MO, USA)	Fresh or cryopreserved
**Medium C**		0.1 mM mercaptoethanol (Pharmacia Biotec)	Cryopreserved
**Medium D**		20 µL/mL ITS 0.1 mM mercaptoethanol (Pharmacia Biotec)	Cryopreserved

**Table 2:** Growth patterns of Triturus cristatus cells. Growth patterns of cells from fresh and cryopreserved media in two and four different media, respectively. The numbers shown indicate the number of replicates.

		**Lost to Infection**		**Growth Patterns**		
	Total no. of replicates	Unsuccessful due to fungus	Unsuccessful due to bacteria	Cell growth not observed	Culture reached 5–100 cells	Cell lines cryopreserved
	**Fresh Tissue in Medium A/B**					
Individual 1	3/3	2/3	0/0	2/0	1/3	0/0
Individual 2	3/3	1/2	0/1	0/1	3/2	1/0
Individual 3	3/3	0/0	0/0	0/0	3/3	0/2
Individual 4	3/3	2/2	0/1	1/1	1/2	0/0
	**Cryopreserved Tissue in Medium A/B/C/D**					
Individual 1	3/3/3/3	1/3/1/1	1/0/0/0	3/3/3/3	0/0/0/0	0/0/0/0
Individual 2	3/3/3/3	0/0/1/1	0/0/0/0	3/1/2/1	0/2/1/2	0/0/0/0
Individual 3	3/3/3/3	2/2/3/1	0/1/2/0	2/3/3/1	1/0/0/2	0/0/0/1
Individual 4	3/3/3/3	2/1/1/2	0/0/0/0	1/3/2/1	2/0/1/2	0/0/0/0

Medias are defined by A, B, C and D, please see Table 1 for more specific information.

**Table 3:** Cell index (CI) obtained for different cell numbers of individual 2 at the three time points. Statistical significance was measured between treatments at various cell numbers for treatments A, B and C and at time points (i), (ii) and (iii) using the xCELLigence^®^ system. Values of *p* < 0.05 are considered to indicate a statistically significant difference.

Time Point	Cell Number	NoMC(A) (µ ± SE)	MC(B) (µ ± SE)	MCPBS(C) (µ ± SE)	One-Way ANOVA	Turkey’s Test
20 h (i)	5000	1.60 ± 0.12	1.37 ± 0.01	1.48 ± 0.13	F = _2.9_ = 1.36, *p* = 0.30	
	7500	3.15 ± 0.13	3.06 ± 0.17	3.03 ± 0.03	F = _2.9_ = 0.23, *p* = 0.79	
	10,000	4.45 ± 0.08	4.60 ± 0.05	4.59 ± 0.05	F = _2.9_ = 1.96, *p* = 0.19	
70 h (ii)	5000	0.85 ± 0.14	0.74 ± 0.05	0.87 ± 0.09	F = _2.9_ = 0.47, *p* = 0.63	
	7500	2.07 ± 0.23	2.10 ± 0.12	1.85 ± 0.14	F = _2.9_ = 0.60, *p* = 0.56	
	10,000	4.47 ± 0.19	5.13 ± 0.09	4.86 ± 0.17	F = _2.9_ = 4.39, *p* = 0.04	(B > A) *
130 h (iii)	5000	0.48 ± 0.19	0.22 ± 0.11	-0.12 ± 0.04	F = _2.9_ = 5.54, *p* = 0.02	(A > C) *
	7500	1.99 ± 0.47	1.34 ± 0.17	0.18 ± 0.07	F = _2.9_ = 10.06, *p* = 0.00	(A > C) **, (B > C) *
	10,000	5.26 ± 0.32	6.42 ± 0.23	3.72 ± 0.36	F = _2.9_ = 18.89, *p* = 0.00	(A > C) *, (B > C) ***

* = *p* < 0.05, ** = *p* < 0.01, *** = *p* < 0.001. Standard error (SE), Treatment A (No media change (NOMC)), Treatment B (Media change (MC)), Treatment C (Media change with PBS (MCPBS)).

**Table 4:** Cell index (CI) obtained for different cell numbers of individual 3 at the three time points. Statistical significance was measured between treatments at various cell numbers for treatments A, B and C and at time points (i), (ii) and (iii) using the xCELLigence^®^ system. Values of *p* < 0.05 are considered to indicate a statistically significant difference. Standard error (SE).

Time Point	Cell Number	NoMC(A) (µ ± SE)	MC (B) (µ ± SE)	MCPBS (C) (µ ± SE)	One-Way ANOVA	Turkey’s Test
20 h (i)	5000	1.61 ± 0.08	1.2 ± 0.08	1.61 ± 0.06	F = _2.9_ = 1.88, *p* = 0.20	
	7500	2.63 ± 0.13	2.16 ± 0.11	2.28 ± 0.07	F = _2.9_ = 5.26, *p* = 0.03	(A > B) *
	10,000	4.90 ± 0.15	5.11 ± 0.13	4.72 ± 0.08	F = _2.9_ = 2.47, *p* = 0.13	
70 h (ii)	5000	1.84 ± 0.08	1.79 ± 0.11	1.69 ± 0.05	F = _2.9_ = 0.75, *p* = 0.49	
	7500	6.18 ± 0.15	6.08 ± 0.35	3.85 ± 0.10	F = _2.9_ = 4.35, *p* = 0.04	(A > C) ***, (B > C) ***
	10,000	6.45 ± 0.37	7.13 ± 0.13	6.86 ± 0.11	F = _2.9_ = 2.11, *p* = 0.17	
130 h (iii)	5000	2.21 ± 0.11	2.24 ± 0.13	1.27 ± 0.03	F = _2.9_ = 30.46, *p* = 0.00	(A > C) ***, (B > C) ***
	7500	3.32 ± 0.12	2.81 ± 0.27	2.25 ± 0.18	F = _2.9_ = 7.11, *p* = 0.01	(A > C) *
	10,000	6.19 ± 0.15	6.08 ± 0.35	3.85 ± 0.10	F = _2.9_ = 33.72, *p* = 0.00	(A > C) ***, (B > C) ***

* = *p* < 0.05, *** = *p* < 0.001. Standard error (SE), Treatment A (No media change (NOMC)), Treatment B (Media change (MC)), Treatment C (Media change with PBS (MCPBS)).

**Table 5:** Cell index (CI) obtained for different cell numbers of individual 3 at three time points. Statistical significance was measured between treatments at various cell numbers for treatments A, B and C and at time points (i), (ii) and (iii) using the xCELLigence^®^ system. Values of *p* < 0.05 are considered to indicate a statistically significant difference.

Time Points	Treatment	Fresh Tissue (µ ± SE)	Cryopreserved Tissue (µ ± SE)	*t*-Test (*p*-Value) = Exact Permutation
20 h (i)	NoMC (A)	4.90 ± 0.15	2.94 ± 0.03	(0.000013) = 0.0142
	MC (B)	5.11 ± 0.13	2.77 ± 0.04	(0.00000) = 0.0142
	MCPBS (C)	4.72 ± 0.08	2.74 ± 0.08	(0.00000) = 0.0142
70 h (ii)	NoMC (A)	6.45 ± 0.37	6.57 ± 0.09	(0.76074) = 0.8285
	MC (B)	7.13 ± 0.13	6.55 ± 0.07	(0.00796) = 0.0142
	MCPBS (C)	6.86 ± 0.11	6.59 ± 0.34	(0.50642) = 0.5857
130 h (iii)	NoMC (A)	6.19 ± 0.15	8.61 ± 0.05	(0.000004) = 0.0285
	MC (B)	6.08 ± 0.35	8.25 ± 0.10	(0.001035) = 0.0142
	MCPBS (C)	3.85 ± 0.10	5.98 ± 0.49	(0.005869) = 0.0285

Standard error (SE), Treatment A (No media change (NOMC)), Treatment B (Media change (MC)), Treatment C (Media change with PBS (MCPBS)).

## Supplementary Materials

The following are available online at https://www.mdpi.com/2076-2615/11/2/367/s1, Table S1: Parameters displaying initial growth patterns; Figure S1: Direct comparison of ideal culture conditions and seeding densities for individual 2; Figure S2: Direct comparison of ideal culture conditions and seeding densities for individual 3.; Figure S3: Direct comparison of growth conditions from a cell line setup from either a fresh (A) or cryopreserved (B) tissue sample from the same individual.
