# Supplementary material for: Establishing Cell Lines from Fresh or Cryopreserved Tissue from the Great Crested Newt (Triturus cristatus): A Preliminary Protocol
Source: Animals (Basel). 2021 Feb 1;11(2):367. doi: 10.3390/ani11020367 (PMC7912844; doi:10.3390/ani11020367)
Supplement: Supplementary file 1 [file animals-11-00367-s001.pdf]

Supplementary information

Table S1. Parameters displaying initial growth patterns. Observed day of growth, day of passage 1 and day of cryopreserving cell line for each replicate of fresh and cryopreserved cultured tissue. - indicate replicates where no growth was observed during the experiment.

		Individual 1	*Individual 2*	*Individual 3*	*Individual 4*
	**Fresh tissue**				
Medium A	Day of growth	18, 67, 26	12, -, 3	16, 39, 18	12, 3, -
	Passage 1			42, 54, 54	
	Day of cryopreservation			93, 153, X	
Medium B	Day of growth	-, 78, -	3, 3, 3	39, 18, 39	-, 3, -
	Passage 1	-	-, 78, -	-, -, -	-
	Day of cryopreservation		109		
	**Cryopreserved tissue**				
edium A	Day of growth		36, -, -		35, -, 28
	Passage 1		-		- -
	Day of cryopreservation				
Medium B	Day of growth			-, 72, 72	
	Passage 1			-, -	
	Day of cryopreservation				
Medium C	Day of growth			-, -, 40	-, 35, -
	Passage 1			-	-
	Day of cryopreservation				
Medium D	Day of growth		37, -, 24	-, 32, 32	35, -, 35
	Passage 1		-, 58	- , -	-, -
	Day of cryopreservation		98		

Figure S1

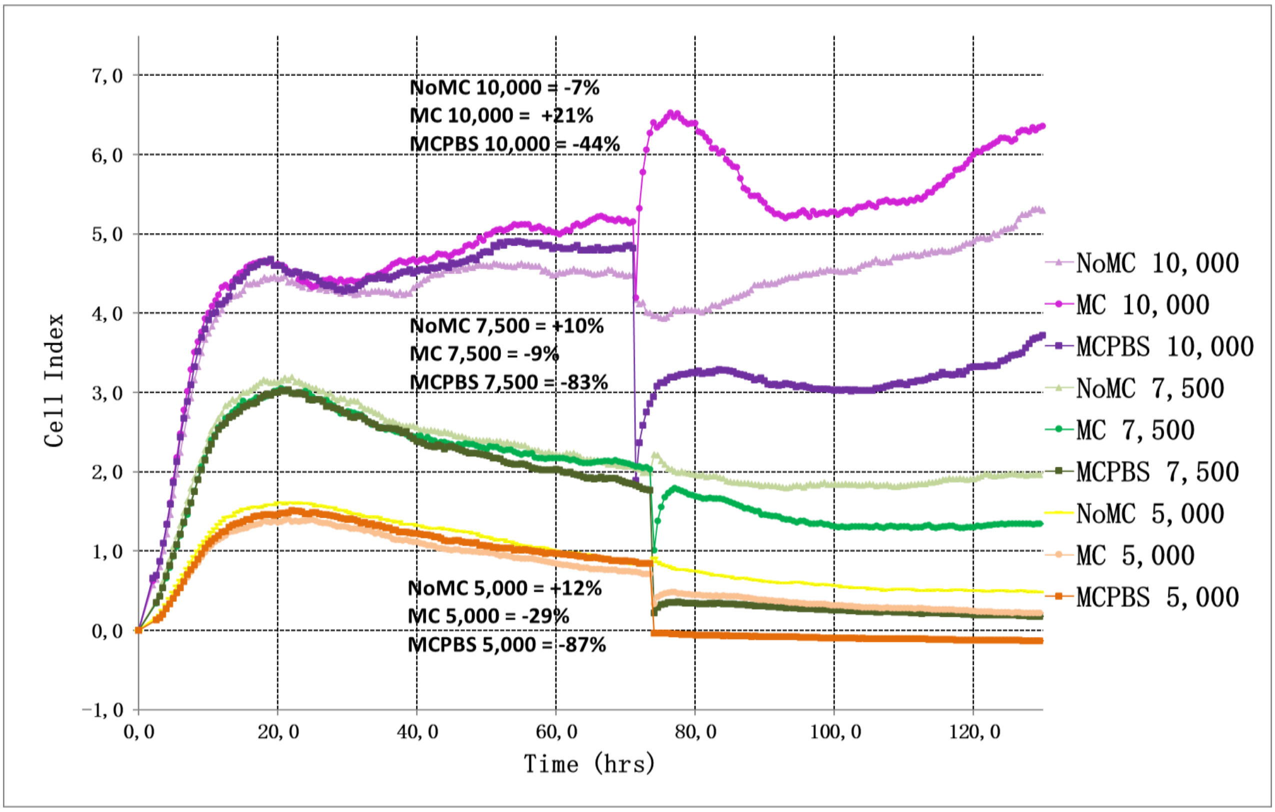

Figure S1: Direct comparison of ideal culture conditions and seeding densities for individual 2. Cell line was set up with media B. Media change was performed after 72 h. Different treatments are displayed by: No media change (NoMC), media change (MC) and media change including washing with PBS (MCPBS). Curves represent the mean cell index value from four replicates. At media change a drop in percentage is calculated and displayed on the graph, (-) indicating a negative impact and (+) indicating a positive impact in percentage. Statistical analyses were performed between treatments and among cell number at 20h (after adherence), 70h (before treatment) and at 130 h (after treatment).

Figure S2

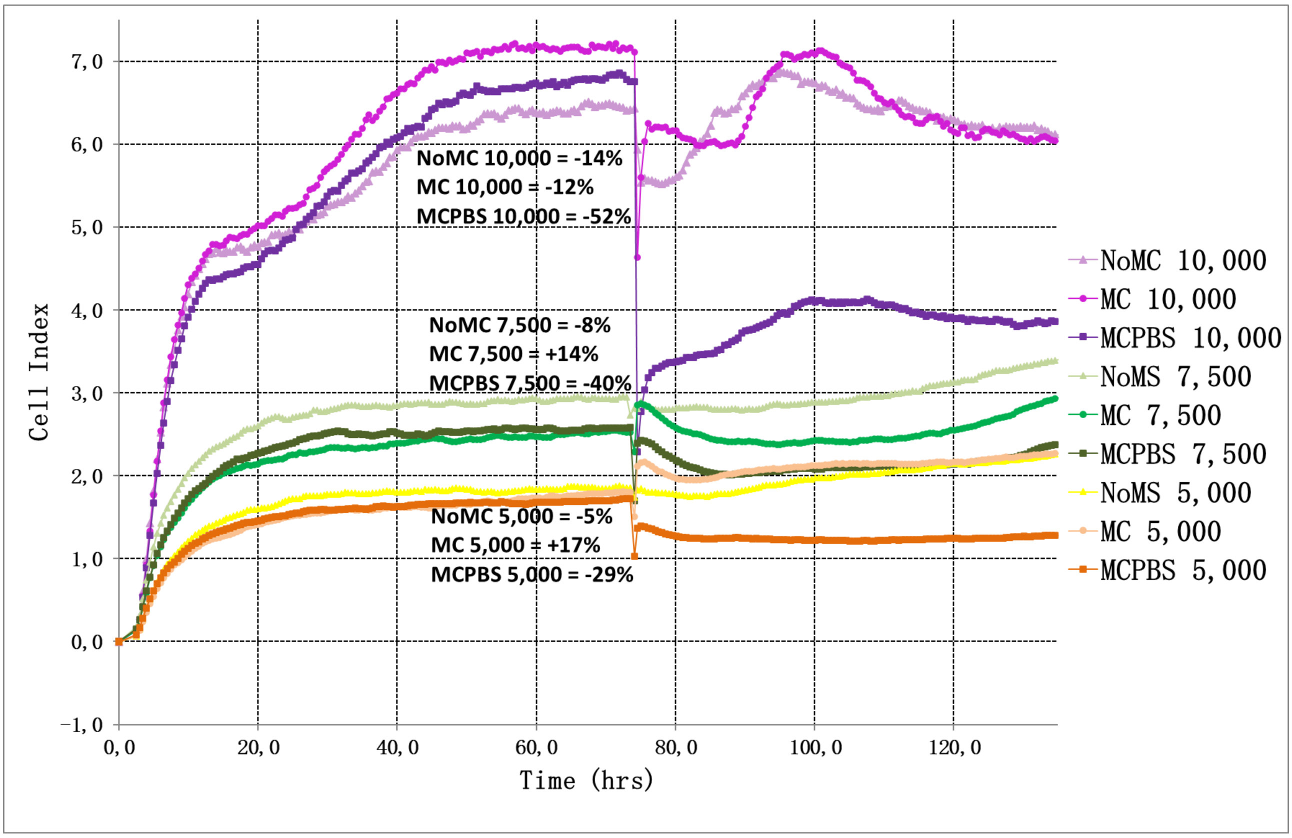

Figure S2. Direct comparison of ideal culture conditions and seeding densities for individual 3. Cell line was set up with media A. Media change was performed after 72 h. Different treatments are displayed by: No media change (NoMC), media change (MC) and media change including washing with PBS (MCPBS). Curves represent the mean cell index value from four replicates. At media change a drop in percentage is calculated and displayed on the graph, (-) indicating a negative impact and (+) indicating a positive impact in percentage. Statistical analyses were performed between treatments and among cell number at 20h (after adherence), 70h (before treatment) and at 130 h (after treatment).

Figure S3

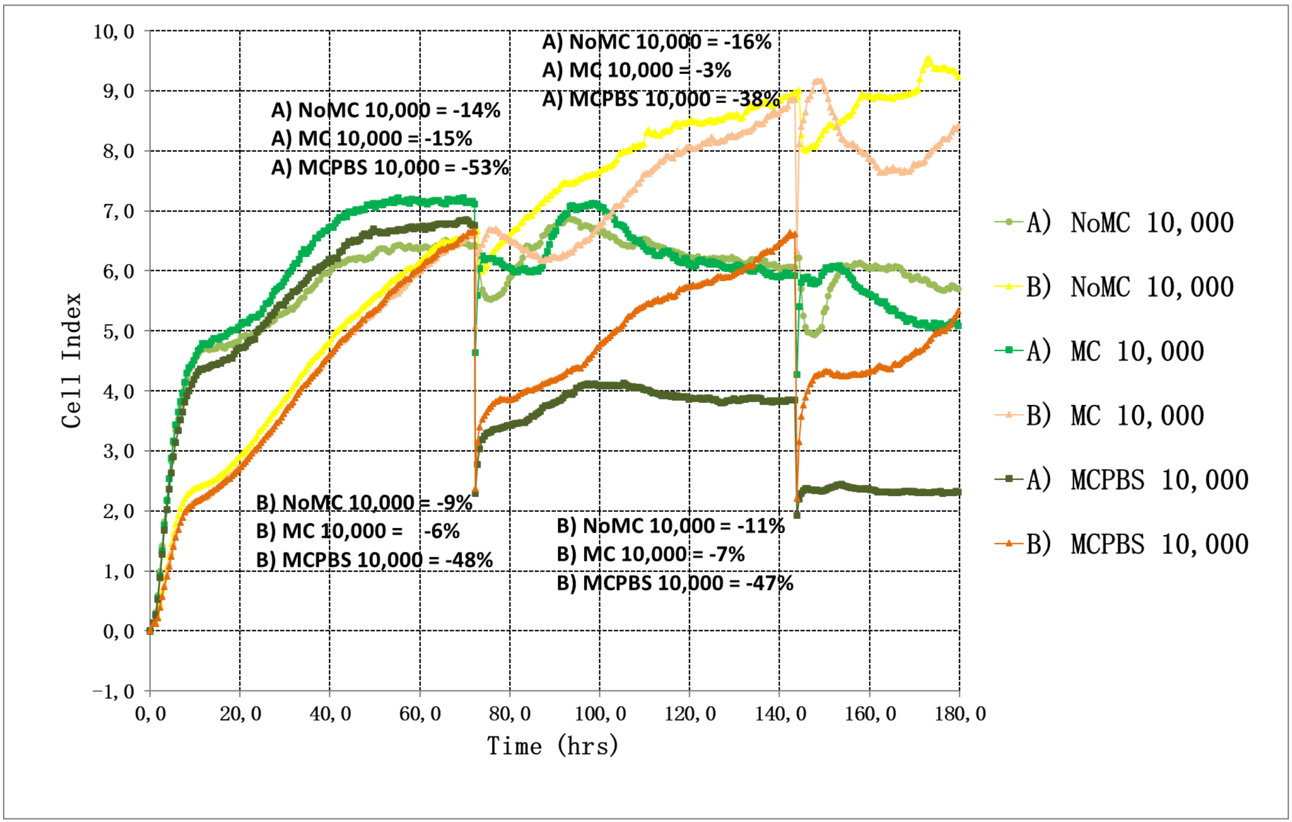

Figure S3: Direct comparison of growth conditions from a cell line setup from either a fresh (A) or cryopreserved (B) tissue sample from the same individual. Cell line A and B were set up with media B and D, respectively. Different treatments were performed at 72 h and 145 h and are displayed by: No media change (NoMC), media change (MC) and media change including washing with PBS (MCPBS). At each media change a drop in percentage is calculated and displayed on the graph, (-) indicating a negative impact and (+) indicating a positive impact in percentage. Statistical analyses were performed between treatments and among cell number at 20h (after adherence), 70h (before treatment) and at 130 h (after treatment).
